# The effects of laryngeal mask airway passage simulation training on the acquisition of undergraduate clinical skills: a randomised controlled trial

**DOI:** 10.1186/1472-6920-11-57

**Published:** 2011-08-11

**Authors:** Elpiniki Laiou, Thomas H Clutton-Brock, Richard J Lilford, Celia A Taylor

**Affiliations:** 1 School of Health and Population Sciences, College of Medical & Dental Sciences, University of Birmingham, Birmingham, UK; 2 School of Clinical and Experimental Medicine, College of Medical & Dental Sciences, University of Birmingham, Birmingham, UK

## Abstract

**Background:**

Effective use of the laryngeal mask airway (LMA) requires learning proper insertion technique in normal patients undergoing routine surgical procedures. However, there is a move towards simulation training for learning practical clinical skills, such as LMA placement. The evidence linking different amounts of mannequin simulation training to the undergraduate clinical skill of LMA placement in real patients is limited. The purpose of this study was to compare the effectiveness *in vivo *of two LMA placement simulation courses of different durations.

**Methods:**

Medical students (n = 126) enrolled in a randomised controlled trial. Seventy-eight of these students completed the trial. The control group (n = 38) received brief mannequin training while the intervention group (n = 40) received additional more intensive mannequin training as part of which they repeated LMA insertion until they were proficient. The anaesthetists supervising LMA placements in real patients rated the participants' performance on assessment forms. Participants completed a self-assessment questionnaire.

**Results:**

Additional mannequin training was not associated with improved performance (37% of intervention participants received an overall placement rating of > 3/5 on their first patient compared to 48% of the control group, X^2 ^= 0.81, p = 0.37). The agreement between the participants and their instructors in terms of LMA placement success rates was poor to fair. Participants reported that mannequins were poor at mimicking reality.

**Conclusions:**

The results suggest that the value of extended mannequin simulation training in the case of LMA placement is limited. Educators considering simulation for the training of practical skills should reflect on the extent to which the *in vitro *simulation mimics the skill required and the degree of difficulty of the procedure.

## Background

The laryngeal mask airway (LMA) can be used in a variety of airway management situations and is in many circumstances an alternative to the more technically demanding process of intubation. It is suggested that effective use of the LMA requires learning proper insertion technique in normal patients undergoing routine surgical procedures with general anaesthesia [1]. However, there is a move towards simulator training, ranging from low- to high-fidelity simulators, for learning practical clinical skills [2-4]. Dierdorf, for example, recommends practice on a mannequin before attempting the technique of LMA insertion on real patients [1]. In this paper we investigated the effectiveness of (and hence necessity for) such training. The ideal study would:

1) Evaluate effectiveness on real patients rather than on the mannequins themselves.

2) Compare outcomes in an intervention group with outcomes in a randomly generated control group.

While numerous studies can be found comparing LMAs to other airway management devices [5-10], the literature investigating the effectiveness of different LMA placement training modalities is limited. In the process of systematically reviewing simulation training we found four studies that had measured outcomes of LMA training *in vitro *(i.e. using a mannequin) but without contemporaneous controls [7,11-13]. These studies fulfilled neither of the above criteria. We found three studies that had measured outcomes on real patients but these did not use contemporaneous controls [14-16] thereby fulfilling only the first of the above criteria. Finally, one study had evaluated LMA placement mannequin training using contemporaneous controls but its outcomes were measured on a cadaver [17]. We found no studies comparing different 'doses' of mannequin training that had fulfilled both our criteria of use of randomised controls and measurement of proficiency on real patients. This paper describes such a study.

Currently medical students at the University of Birmingham in the UK receive only very limited instruction on LMA placement and the majority have one or two practice attempts on a mannequin. This study set out to test the hypothesis that an additional session of formal simulation training would promote speed of learning and result in a higher level of skill than brief simulation training when LMA placement is first undertaken in clinical practice. An additional objective was to compare self-assessment of success in this particular procedure with assessment by a third party in a clinical setting.

## Methods

### Participants

The study was approved by the West Midlands Multi-Centre Research Ethics Committee and was undertaken during the period August 2006 to March 2007. Eligible participants were all Year 4 University of Birmingham medical students who were about to undergo their clinical attachment in Anaesthesia, Respiratory and Intensive Care Medicine (ARICM) for the academic year 2006-2007. One hundred and twenty-six students volunteered to take part after listening to a talk describing the study and each participant gave written consent. Participants completed a brief baseline questionnaire recording their demographic features and any previous LMA placement training that they had received. All participants had received the standard brief mannequin training in LMA placement that is a compulsory part of their ARICM module. This typically involves a demonstration of the technique by a clinical instructor on a mannequin followed by one or two attempts by the trainees and lasts around 5 minutes.

### Baseline assessment

Each participant undertook a baseline assessment at The University of Birmingham Medical School. This consisted of performing LMA placement on a mannequin (Laerdal Adult Airway Trainer) once using a size-4 LMA Classic™. Two anaesthetists rated each student's performance on a pro-forma. The time to ventilation success or failure was recorded. Ventilation success was verified by direct visualisation of chest expansion of the mannequin with bag-tube-ventilation. In the cases where the two raters did not agree on ventilation success, the attempt was recorded as unsuccessful. The anaesthetists rated the participants' handling of the LMA and their overall success in LMA placement using a 5-point Likert-type scale. Following the baseline assessment, each participant was asked to open an envelope containing their group allocation. Block randomisation and sequentially numbered sealed opaque envelopes were used for the allocation [18].

### Intervention

Participants in the control group received no additional mannequin training. Participants in the intervention group received approximately 20 minutes of additional LMA placement training on Laerdal Airway Management Trainers, administered at the end of the baseline assessments. The participants were taught the use of the LMA (size 4, LMA Classic™) on the mannequin in groups of 4-8. The training consisted of a step-by-step demonstration of the index finger LMA insertion technique (Table 1) on the mannequin by an anaesthesiologist and supervised practice of the technique until the participants had demonstrated a correct mannequin LMA placement. Training was in accordance with the instruction manual [19].

**Table 1:** The steps of the LMA insertion technique

1	Tightly deflate the cuff with a syringe
2	Lubricate the posterior surface of the cuff
3	Place yourselves behind the patient
4	Push their head with the non-dominant arm to achieve extension of their head with flexion of the neck
5	Hold the LMA like a pen, with the index finger placed at the junction of the cuff and the airway tube
6	Press the tip of the cuff upward against the hard palate and flatten the cuff against it
7	Push the jaw downward with your middle finger
8	Using the index finger, advance the device into the hypopharynx until resistance is definitely felt
9	Press down on the tube with your non-dominant hand
10	Remove the index finger
11	Inflate the cuff with up to 30 mL of air without holding the tube
12	Connect a self-inflating bag to the tube and ventilate the lungs

Four instructors conducted the training; one was a senior Consultant in Anaesthesia and Critical Care with more that 15 years of teaching experience. The other three, who worked under his supervision, were senior medical trainees in anaesthesia, all with previous experience of teaching practical procedures to medical students. All instructors were experienced in LMA placement. Uniformity of teaching was ensured by a common session at the beginning rehearsing the teaching material and by everyone following the step-by-step teaching approach described in the LMA airway instruction manual (Table 1).

Twenty minutes was chosen as the duration for the intervention training based on the recommendation of the senior Consultant in Anaesthesia and Critical Care who was also the Lead of the ARICM module. This was due to the practical considerations that would have arisen had it been found worthwhile to implement the additional training for all students.

### Clinical practice assessment

All participants were given a pack containing four sequentially numbered assessment forms in sealed envelopes, a clinical practice self-assessment questionnaire and written instructions on how to fill these in. Participants subsequently spent six weeks undertaking their standard ARICM clinical training in one of 11 West Midlands hospitals. The clinical instructors supervising the participants' LMA placements in the operating room were asked to fill in an assessment form on the participants' first four attempts to insert an LMA. The patients were selected by the clinical instructors and were American Society of Anaesthesiologists' (ASA) class 1 or 2 adults undergoing routine surgical procedures under general anaesthesia. These were patients in whom a difficult airway was not anticipated. The interval between the additional mannequin training and these assessments ranged from two days to six weeks.

The form contained questions about placement success and the following data were collected: 1) rating of overall success (the primary outcome) of the LMA placement on a 5-point Likert-type scale (1 being 'extremely poor' and 5 being 'excellent') 2) successful ventilation following the LMA placement, 3) rating of the handling of the LMA during the insertion on a 5-point Likert-type scale, 4) whether time to successful placement was less than 40 seconds and 5) the number of insertion attempts. Successful ventilation was determined clinically by observation of adequate seal, satisfactory chest movement and/or observation of a normal capnographic curve where applicable. The instructors conducting the assessments were 'blind' to group assignment and the participants had been asked not to reveal their group allocation.

The participants were also asked to fill in a self-assessment questionnaire on their first 4 LMA placements in patients. The questionnaire asked participants to rate their own handling of the LMA and their overall success in LMA placement for each patient on 5-point Likert- type scales identical to those used by their instructors. It also asked them to comment on the usefulness of their respective mannequin training.

### Sample size

Based on consultation with the Year 4 Lead of the ARICM module, the probability of receiving an overall LMA placement success rating of > 3 (i.e. above average) on the corresponding Likert-type scale, for a student's first patient, was set at 0.50 for the control group and at 0.75 for the intervention group. Based on 80% power to detect a statistically significant difference (α = 0.05, one-sided), 46 participants were required for each study group. A one-sided test was used as it was hypothesised that the additional simulation training would not result to poorer skill than brief simulation training. To compensate for drop-out/failure to complete, the planned number of participants had been 85 participants per group.

### Analysis

Statistical analysis was performed using SPSS 12.0.1 and R 2.6.2 for Windows. The distribution of data was determined using the Shapiro-Wilk test of normality. Pearson Chi-squared analyses were used to compare groups in terms of rates of being given an overall performance rating of > 3, achieving effective ventilation, establishing ventilation in ≤ 40 sec and achieving effective ventilation at the first insertion attempt. Fisher's Exact Test was used when expected counts were less than 5. Fisher's randomization T-test was used to compare groups in terms of LMA handling and overall LMA placement success ratings of 1-5. The Mann-Whitney U test was used to compare successful attempt times in the baseline assessment as data were not normally distributed. A p-value of < 0.05 would be considered statistically significant. The relationship between instructor and participant overall LMA placement success ratings was analysed using the Spearman's ρ (rho) correlation coefficient. Clinical practice instructor-participant agreement with regards to whether they awarded an overall LMA placement success rating of < 3, 3 or > 3 were explored using the kappa statistic [20]. Pair wise deletion was used with regards to missing data. The participants' quotes on the self-assessment questionnaire were categorised into common themes.

## Results

### Participants

**Figure 1:**
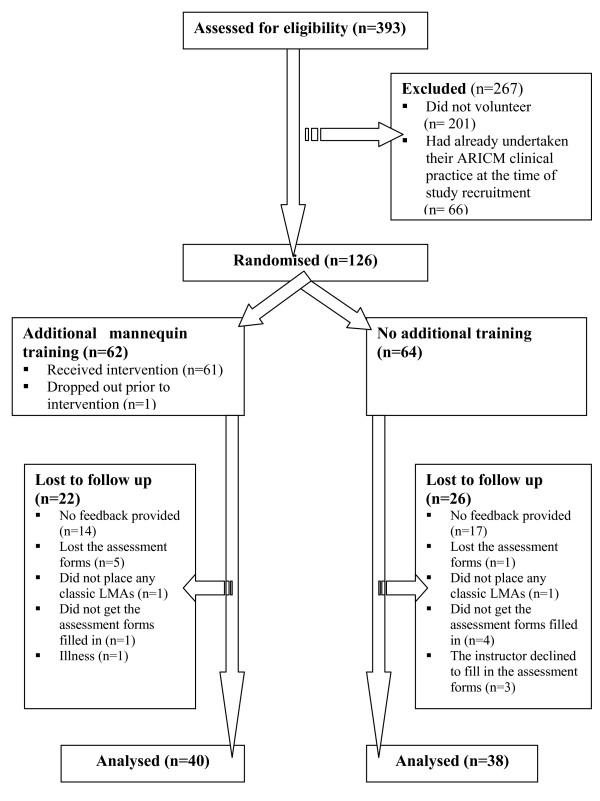
**Participant flow chart following the CONSORT scheme**.

One hundred and twenty six Year 4 medical students (32% of the whole Year 4 population) enrolled in the study and participated in the baseline assessment (Table 2). Overall, 78 (62%) of the participants who enrolled completed the study by returning their instructors' assessment forms following their clinical attachment (Figure 1). Four (5%) of the participants who completed the study were Graduate Entry Course (GEC) students i.e. they had previously obtained a First or 2:1 Honours degree in a life sciences discipline. Three additional participants returned their clinical practice self-assessment questionnaires, giving a total of 81 (64% of those enrolled) participants who returned some form of data at the end of the study. The study population and the overall MBChB Year 4 population were similar in terms of mean age and nationality and the differences in terms of sex and percentages of GEC students were small (Table 3).

**Table 2:** Participant characteristics at baseline and follow-up

	At baseline		At follow-up**	
	
Study group	Intervention	Control	Intervention	Control
**No of participants**	62	64	40	38

**Age mean **(SD) (years)	22.6 (1.6)*	22.4 (1.2)	22.7 (1.7)	22.3 (1.0)

Sex:Male	20 (32%)	23 (36%)	11 (28%)	12 (32%)
Female	42 (68%)	41 (64%)	29 (72%)	26 (68%)

**Ethnicity**:				
British White	44 (71%)	37 (58%)	31 (77%)	22 (58%)
British Asian	9 (14%)	11 (17%)	4 (10%)	6 (16%)
British other	2 (3%)	6 (9%)	0 (0%)	4 (10%)
Non British	6 (10%)	10 (16%)	4 (10%)	6 (16%)
Not disclosed	1 (2%)	0 (0%)	1 (3%)	0 (0%)

**Prior LMA practice:**				
Mannequin ARICM	43 (69%)	43 (67%)	28 (70%)	28 (74%)
Other mannequin	3 (5%)	4 (6%)	2 (5%)	3 (8%)
Patient	7 (11%)	12 (19%)	4 (10%)	8 (21%)
None	17 (27%)	15 (23%)	11 (28%)	6 (16%)

**GEC students:**	3 (5%)	3 (5%)	3 (8%)	1 (3%)

*N = 61

**Returned instructors' assessment forms

**Table 3:** Participant characteristics at follow-up versus overall MBChB Year 4 population characteristics

Group	Study participants	Overall population
**No of participants**	78	393

**Age mean (SD) years**	23.2 (1.3)	23.4 (1.8)

**Sex:**		
Male	23 (29%)	158 (40%)
Female	55 (71%)	235 (60%)

**Nationality:**		
British	68 (87%)	358 (91%)
Non British	10 (13%)	35 (9%)

**GEC students**	4 (5%)	42 (11%)

### Baseline assessment

All 126 participants (100%) had one attempt in LMA placement on a mannequin. Eighty-three (66%) achieved successful ventilation. No statistically significant differences were found between groups at baseline in terms of overall success mean ratings, successful ventilation, LMA handling mean ratings and time to successful ventilation (Table 4).

**Table 4:** Baseline assessment success rates and successful ventilation times

	All participants (N = 126)				Participants followed-up (N = 78)			
	I*	C*	**Test** **statistic**	**P-value**	**I**	**C**	**Test** **statistic**	**P-value**

Overall success rating > 3	22.6%	18.8%	X^2^ 0.282	0.595	25.0%	18.4%	X^2^ 0.495	0.482

Overall success (mean rating)	2.88	2.70	-** 0.18	0.172^┼^	2.98	2.68	- 0.30	0.122^┼^

Successful ventilation	62.9%	68.8%	X^2^ 0.479	0.489	67.5%	65.8%	X^2^ 0.026	0.873

LMA handling (mean rating)	3.02	2.98	- 0.04	0.453^┼^	3.10	3.05	- 0.05	0.450^┼^

Time to task (SD) (sec)	33.6 (12.2)	32.3 (13.2)	Mann- Whit. U 787.5	0.519	33.9 (12.7)	29.1 (11.7)	Mann- Whit. U 262.0	0.165

* I = Intervention group, C = control group

**- = difference between means

^┼ ^P-value obtained using Fisher's randomisation t-test

### Clinical practice

The primary outcome was instructors' Likert-type ratings of overall LMA placement success in the first patient (Table 5). There was no difference in the percentage of participants achieving a rating of > 3 between the intervention and control groups (37% Vs. 48%, X^2 ^= 0.81, p = 0.37).

**Table 5:** Instructor outcome measures during clinical practice

		Intervention (I)	Control (C)	Test statistic	P-value
**1st Patient**	Overall success rating > 3	37.1% (n = 35)	48.3% (n = 29)	X2 0.806	0.369
	
	Overall success (mean rating)	3.34 (n = 35)	3.55 (n = 29)	-* -0.21	0.168^┼^
	
	Successful ventilation	82.9% (n = 35)	96.6% (n = 29)	Fisher's exact test	0.116
	
	LMA handling (mean rating)	3.23 (n = 35)	3.40 (n = 30)	- -0.17	0.168^┼^
	
	Time to task ≤ 40 sec	74.3% (n = 35)	72.4% (n = 29)	X2 0.028	0.866
	
	Success at 1^st ^attempt	66.7% (n = 33)	74.1% (n = 27)	X2 0.388	0.533

**4th Patient**	Overall success rating > 3	58.8% (n = 34)	63.6% (n = 33)	X2 0.163	0.686
	
	Overall success (mean rating)	3.68 (n = 34)	3.70 (n = 33)	- 0.02	0.500^┼^
	
	Successful ventilation	85.7% (n = 35)	93.9% (n = 33)	Fisher's exact test	0.429
	
	LMA handling (mean rating)	3.65 (n = 34)	3.73 (n = 33)	- -0.08	0.399^┼^
	
	Time to task ≤ 40 sec	85.7% (n = 35)	84.4% (n = 32)	X2 0.024	0.878
	
	Success at 1^st ^attempt	79.3% (n = 29)	84.8% (n = 33)	X2 0.324	0.569

*- = difference between means

^┼ ^P-value obtained using Fisher's randomisation t-test

No significant differences were found between the groups in achieving successful ventilation in real patients and the trend was in the direction of more success among controls (Table 5). The main reasons for failed LMA placement attempts included inadequate seal, failure of the participant to position the LMA and patient-related factors such as difficult airways, light anaesthesia or edentulous patients.

No statistically significant differences were found between the groups in the instructors' Likert-type ratings of LMA handling during the insertion, achieving effective ventilation in ≤ 40 sec or achieving ventilation in at 1^st ^insertion attempt (Table 5). Although not a direct objective of this study, it is notable that both groups improved across all outcomes between their 1^st ^and 4^th ^patient (Table 5).

### Participant self-assessments

**Figure 2:**
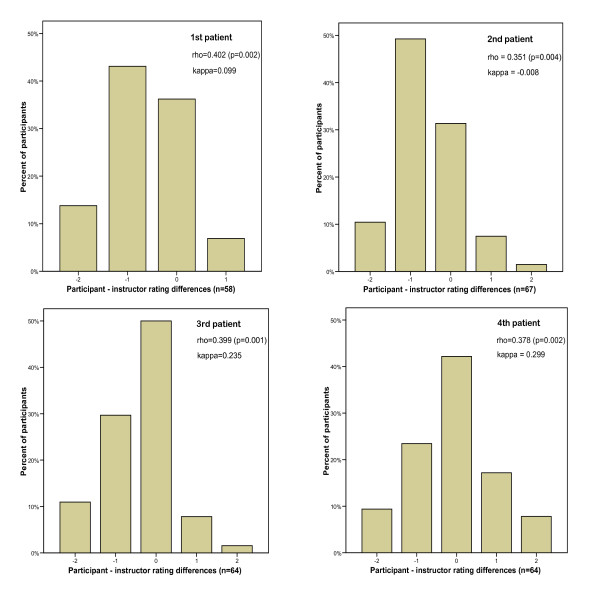
**Differences between participant self-ratings and instructor ratings for overall success of LMA placement (difference = participant rating - instructor rating)**.

When comparing the participants' self-ratings of overall LMA placement success to the same ratings by their instructor, participants tended to underrate their first two LMA placements (Figure 2). The correlation between instructor and participant ratings was moderate (Figure 2). The agreement between individual participants and their instructors in terms of whether they had achieved an overall LMA placement success score of < 3, 3 or > 3 was poor during the first two assessments and fair during the third and fourth assessments.

### Participant feedback

Seventy-two participants (57% of those enrolled in the study) returned their self-assessment clinical practice questionnaires. Sixty nine percent of the questionnaire respondents 'agreed' or 'strongly agreed' that their overall mannequin training had been helpful, 14.1% 'disagreed or strongly disagreed', 9.7% were 'undecided' and 5.6% indicated that they had not had any mannequin practice. The latter had been in the control group. There was no statistically significant difference between the two groups in terms of the number of participants who 'agreed' or 'strongly agreed' that their mannequin training was helpful (X^2 ^= 2.26, p = 0.13).

Fifty-seven of the respondents opted to include some further qualitative feedback. Twenty-four respondents (42% of those providing qualitative feedback) felt that the mannequins mimicked reality poorly. Nineteen respondents (33%) reported that mannequin training had helped them to learn the basic technique required in order to place an LMA before approaching any patients. Eight respondents (14%) reported that mannequin training had made them more confident.

## Discussion

### Summary of findings

The purpose of this study was to investigate whether a period of additional mannequin training, added to a very basic exposure of LMA placement on mannequins, would increase medical students' LMA placement success during their regular clinical practice with real patients. The participants who received the additional mannequin training session achieved similar success rates in placing LMAs to those who received only one brief mannequin training session across all outcomes studied. Overall, participants tended to underrate their performance for the first two LMA placements and agreement between participant ratings and their instructors' ratings was generally poor to fair. Participants' perceptions of the value of their mannequin training did not differ significantly between the intervention and the control group. According to their qualitative feedback, mannequins were poor at mimicking reality.

### Relationship to previous studies

The current study findings suggest that merely increasing the 'dose' of mannequin practice yields little or no additional benefit. Our results broadly corroborate the only other controlled study of LMA placement training that assessed effectiveness of different 'doses' of mannequin training [17]. In this study the performance (in a cadaver) of a group of dental students who had practiced 10 times on the mannequin was not statistically significantly different in terms of placement grades from a group who had practiced 5 times. Nevertheless, in contrast to our findings the trend was in the direction of improved performance with more opportunity to practice.

Davies et al [14] trained 11 naval medical trainees using videotapes, mannequin practice and a demonstration on an anaesthetised patient. They reported 100% success in their participants' first patient in terms of LMA insertion, 82% success for the second patient, above 90% subsequently and an overall success rate of 94%. Their first patient success rates are higher than in this study. However, the choice of patient where trainees were tested on was more controlled i.e. ASA 1 patients only and their training intervention included multiple instruction modalities. Overall, their success rates seem to be comparable to the current study.

Additionally, Roberts et al [21] compared mannequin training only (five LMA insertions) to the same mannequin training plus additional *in vivo *training with patients. They found a 75% first attempt LMA insertion success rate *in vivo *in their 'mannequin training only' group. This was higher compared to the intervention group's first attempt success rate in this study, yet it was comparable to the first attempt success rate of this study's 'brief mannequin training only' control group.

### Consideration of possible mechanisms and explanation

The current study findings suggest that merely increasing the 'dose' of mannequin practice past an initial brief session leads to no additional benefit, at least when this involves low-fidelity mannequins. Three (non-exclusive) reasons might explain the null results of this study. Firstly, the training offered may have been sub-optimal. However, in a recent systematic review repetitive practice and providing feedback were the top two features acknowledged as important for effective learning in medical simulations [21] and these tenets of good practice were followed in the intervention training. Conversely, a recent systematic review of comparative studies of clinical skills training that focused on intubation, venous cannulation and central venous line insertion concluded that the addition of simulators, including mannequins, to a traditional course was not supported by study results [22].

A second possibility is that the mannequins were too basic. They consequently failed to provide some other important features identified in Issenberg et al's review; providing a range of difficulty levels, capturing clinical variation and providing high degrees of realism [21]. Many of our participants drew attention to the rather low fidelity of the simulators. Furthermore, simulators cannot mimic the existential experience of clinical practice, especially in the atmosphere of an acute setting such as an operating theatre. The improvement observed over only four patients in real practice settings adds credence to this explanation.

Our third explanation rests on the observation that LMA placement is a relatively simple technical skill to acquire [23] - this is in large measure the reason behind the introduction and rapid dissemination of this method of airway management [7]. However, first attempt LMA placement success rates lower than 80% have been reported in the literature [24-26], suggesting that additional training, particularly for students with little clinical exposure, is required. This observation leads to a hypothesis for further testing: the need for simulation training increases with the degree of difficulty of the task. Below a certain difficulty threshold, it may be more cost-effective to train 'at the bedside', with minimal possible risk to patients.

### Study limitations

We used a single type of laryngeal mask airway, the LMA Classic™ in this study. There are now many different types of LMA available for use. These devices have three main components (airway tube, mask and inflation line). Most of the LMA insertion techniques follow the same principle and LMA placement remains part of the airway management skills taught to undergraduate medical students. Thus, we feel that the conclusions of this study are also valid to the majority of LMA models in current use.

Ethical considerations prevented including a no practice group in this study as mannequin practice in LMA placement is a set part of the 4^th ^year medical student curriculum. In addition, the length of the extended simulation training provided to the intervention group was only 20 minutes. However, LMA placement is only one of several practical skills taught to 4^th ^year medical students; an intervention of greater length would not have been feasible to integrate in practice into a full curriculum. While the training time per student could be viewed as short, it was also intensified as the whole session was solely focused in LMA placement and participants continually received real-time feedback by their instructors.

A large number of different instructors took part in the assessment and they had no specific training in evaluating proficiency for this study. The baseline data showed that the rater agreement in five different pairs of assessors using the Likert type scales of overall LMA placement success and LMA handling during the insertion ranged from poor to fair although agreement between the different groups of assessors ranged from moderate to very good in the assessment of adequacy of ventilation by direct visualisation of the mannequin. It has been shown that clinical assessment of correct LMA placement based on a perception of unimpaired air-movement does not always lead to identifying LMA malposition [27]. Fiberscopy after insertion of the LMA is recommended as a useful way of assessing the mask position as the LMA can allow adequate ventilation even if sub-optimally placed [28]. However, fiberscopy was not routinely available in the hospitals taking part in the study or used by any of the instructors. Failure to achieve an adequate seal for ventilation with a self-inflating bag was easily detected however it was not possible to reliably assess small leakages as part of the LMA placement success.

There was a large number of MBChB Year 4 students who did not volunteer for the study. It could be argued that this may have led to sampling bias due to our study group being highly motivated. Great effort was made to recruit as many participants as possible, including email advertising, prize draws and a talk inviting them to participate. Afterwards, we compared our study participants to the overall Year 4 population; They were similar in terms of mean age and nationality and the difference in terms of sex was small.

A significant number of participants were lost to follow-up. This led to the intervention and control group in this study having fewer participants than deemed necessary by our original sample size calculation, which was 46 per group. This should be taken into account when evaluating the results of this study.

In addition, low response rates can lower the chance of getting similar groups in terms of key characteristics. However, these losses did not seem to have imbalanced the characteristics of the participants from baseline to follow-up. A similar number of participants dropped out from both the intervention and control groups.

Incomplete data among both participants and their instructors also limited the analysis. As a result in many cases data on individual attempts were missing completely or insufficient information was provided on reasons for failure and exact time to insertion. Prior to the study it was thought that LMA placement was an invariable component of ARICM training. It transpired that some students do not get to practice LMA placement as much as four times. Furthermore, there were cases were students lost the forms or forgot to take them to the operating room.

### Clinical and research implications

A number of participants' comments indicated that the lack of realism of the mannequins limited their training effect. These comments seem to complement the findings of this study and carry four main implications. Firstly, the basic mannequin airway significantly differs from the human airway in important aspects and the clinical context is far removed from the training environment. The users should be made aware by their instructors of these aspects and of clinical variations encountered in real life. Secondly, future research should seek to compare the basic mannequins currently used for training to training on high-fidelity mannequins that provide a range of difficulty levels, and to assess whether this latter training would be more effective in preparing students for real life practice. Thirdly, great care should be exercised in interpreting self-rated assessments and they should not be used as a surrogate for observations of clinical proficiency. Fourthly, we should test the hypothesis that it may be more cost-effective in terms of training outcomes to train under supervision 'at the bedside' for technical tasks such as LMA placement that have a low degree of difficulty at the initial stages of learning.

## Conclusion

The results of this study suggest that the value of extended low-fidelity mannequin training in the case of LMA placement is limited. Educators considering simulation for the training of practical skills should reflect on the extent to which the *in vitro *simulation mimics the skill required and the degree of difficulty of the procedure. Future research should test the hypothesis that the need for simulation training increases with the degree of difficulty of the task. For technical tasks that are below a certain difficulty threshold, it may be more cost-effective to train 'at the bedside'.

## Competing interests

The authors declare that they have no competing interests. The authors alone are responsible for the content and writing of this article.

## Authors' contributions

EL participated in the design, coordination, implementation and statistical analysis of the study and in manuscript preparation and revision. CAT participated in the design, implementation and review of the statistical analysis of the study and in manuscript preparation and revision. TCB participated in the design, coordination, implementation of the study and in manuscript revision. RJL participated in the design of the study, review of the data and in manuscript preparation. All authors read and approved the final manuscript.

## Pre-publication history

The pre-publication history for this paper can be accessed here:

http://www.biomedcentral.com/1472-6920/11/57/prepub
